# Characterisation of a low methane emission rice cultivar suitable for cultivation in high latitude light and temperature conditions

**DOI:** 10.1007/s11356-023-28985-w

**Published:** 2023-07-27

**Authors:** Jia Hu, Mathilde Bettembourg, Silvana Moreno, Ai Zhang, Anna Schnürer, Chuanxin Sun, Jens Sundström, Yunkai Jin

**Affiliations:** 1 Department of Plant Biology, Sweden University of Agricultural Science, The Linnean Centre for Plant Biology, Box 7080, SE-75007 Uppsala, Sweden; 2 Department of Molecular Sciences, Swedish University of Agricultural Sciences, Box 7015, SE-750 07 Uppsala, Sweden

**Keywords:** Global warming potential, Rice cultivation, Methane emission, Heavy metals, High latitude, Sustainable agriculture

## Abstract

Rice cultivation on paddy soil is commonly associated with emissions of methane, a greenhouse gas, but rice varieties may differ in their actual level of emissions. This study analysed methane emissions associated with 22 distinct rice genotypes, using gas chromatography, and identified the cultivar Heijing 5 from northern China as a potential low-methane rice variety. To confirm this and to examine whether Heijing 5 can perform similarly at higher latitudes, Heijing 5 was cultivated in field trials in China (lat. 32° N) and Sweden (lat. 59° N) where (i) methane emissions were measured, (ii) methanogen abundance in the rhizosphere was determined using quantitative PCR, and (iii) the concentrations of nutrients in water and of heavy metals in rice grain and paddy soil were analysed. The results demonstrated that the low-methane rice cultivar Heijing 5 can successfully complete an entire growth period at high-latitude locations such as central Sweden. Massively parallel sequencing of mRNAs identified candidate genes involved in day length and cold acclimatisation. Cultivation of Heijing 5 in central Sweden was also associated with relatively low heavy metal accumulation in rice grains and lowered nutrient losses to neighbouring water bodies.

**Supplementary Information:**

The online version contains supplementary material available at 10.1007/s11356-023-28985-w.

## Introduction

Rice cultivation contributes significantly to greenhouse gas emissions in the form of methane, with an estimated ~ 11% of atmosphere methane deriving from rice paddies (Jiang et al. 2017), making it the fourth largest contributor after wetlands (22%), coal mining (19%), and enteric fermentation (16%) (https://www.upsbatterycenter.com/blog/greenhouse-gases-methane-causes/). The methane emitted from rice fields is produced by methanogenic archaea that thrive under anaerobic conditions in the rice rhizosphere (Liesack et al. 2000). From the rhizosphere, the methane diffuses into the root air spaces and is released into the atmosphere through the aerenchyma (Kim et al. 2018; Philippot et al. 2011). It is possible to influence methane emissions through different management practices such as water and fertiliser management and biochar application (Malyan et al. 2016; Jiang et al. 2019). Choice of rice variety has also been suggested as possible means to reduce methane emissions from rice cultivation, and molecular breeding tools have been employed to produce individual rice lines associated with drastically reduced (> 90%) methane emissions (Su et al. 2015; Jiang et al. 2017; Du et al., 2021). These transgenic lines allocate more carbon to aboveground tissues, giving higher yield, while less carbon is released from the rice roots to the rhizosphere soil in exudates, reducing the available substrate for methanogenic archaea.

Another environmental problem with rice cultivation is that excessive fertilisation can cause enrichment of macronutrients (i.e. nitrogen (N), phosphorus (P), and potassium (K)) and organic matter in surrounding water bodies (Tusseau-Vuillemin 2001; Kobetičová and Černý 2019). In combination, excessive fertilisation and the intense irrigation associated with rice cultivation can increase the risk of nutrient losses from the rootzone to groundwater (percolation losses) (Chen et al. 2022; Amin et al. 2021; Li et al. 2021a). In addition, the soils used for rice production in many of the major rice-producing countries in the world are contaminated by heavy metals such as arsenic (As), lead (Pb), cadmium (Cd), mercury (Hg), and chromium (Cr) (Ahad et al. 2017). This is a result of accumulation throughout years of sludge application, wastewater irrigation, fertiliser application, and atmospheric deposition (Li et al. 2014; Palansooriya et al. 2020; Khan et al. 2021). Heavy metal accumulation is recognised as a significant environmental problem that threatens rice production, food safety, and human health (Goncalves et al. 2012; Zhao et al. 2015; Rahman et al. 2018). For example, during the past decade, 30–60% of the population of Bangladesh have been exposed to concentrations of As that pose an elevated risk of developing different forms of cancer, including lung, bladder, and skin cancer (Smith et al. 2000).

Some initiatives have been taken recently to breed rice varieties that are metal-tolerant and do not accumulate heavy metals in the rice grains (Haider et al. 2023). QTL mapping and genome-wide association studies (GWAS) have identified genomic regions that make rice tolerant to (and accumulate less) heavy metals such as Cd and As (Murugaiyan et al. 2019; Pan et al. 2020). While plant breeding for such complex traits is difficult and time-consuming, the environmental problems associated with rice cultivation urgently need to be addressed since global demand for rice products will likely increase in the future.

Rice currently accounts for 21% of total global food energy intake (Gayen et al. 2016) and even if that percentage remains unchanged demand will increase because of the predicted global population expansion from 7.8 billion in 2020 to 9.9 billion by 2050 (Chen et al. 2014). Rice is produced in many parts of the world, but the leading producing countries are China and India, which account for more than 50% of global rice production (Food and Agricultural Organization of the United Nations (FAO) 2019). In Europe, rice is mainly produced in the Mediterranean countries (Spain and Italy), while rice cultivation in northern Europe is limited because available commercial rice varieties are not adapted to the short growing season, long daylight conditions, and low temperatures of high-latitude regions (GRiSP 2013). Nevertheless, rice consumption in Europe has increased greatly during the past decade, even in northern Europe (Salamon et al. 2017).

This study addressed three main environmental concerns commonly associated with rice cultivation: (i) methane emissions, (ii) nutrient losses to surrounding water bodies, and (iii) accumulation of heavy metals in rice grains. First, 22 local rice varieties or breeding lines were screened for low methane emissions. This screening step identified a japonica rice variety (*Oryza sativa* ssp. *Japonica* var. Heijing 5) associated with relatively low methane emission rates. Heijing 5 is commonly grown in China at latitudes 43–50° N, and a small-scale pilot study conducted in 2019 indicated that it can be cultivated at higher latitudes (Fei et al. 2020). To confirm this, field experiments with the Heijing 5 variety were performed in China (at latitude 32° N) and in central Sweden (at latitude 59° N). Field evaluations included determination of yield potential and analyses of methane emissions, nutrients in water, and heavy metals in soil and rice grains. In cultivation at the Swedish site, nutrient-rich water from the nearby river Fyris was used for irrigation and fertilisation. Compared with soils in southern Europe and Asia, Swedish soils have a low content of many heavy metals (Toth et al. 2016; Hu et al. 2020). Therefore, this study evaluated whether extending the cultivation zone of rice to high-latitude countries could be a way to reduce nutrients in water and produce rice grains with low levels of heavy metals. To identify possible genetic mechanisms underlying adaptation of Heijing 5 to high-latitude growth conditions, the transcriptome of field-grown plants at later tillering stage was analysed and possible candidate genes connected to flowering time and adaptation to cold temperatures stress were identified.

The overall aim of this work was to evaluate whether a rice variety associated with low methane emissions can be cultivated in high-latitude countries such as Sweden and whether cultivation in this region could help alleviate some of the major environmental concerns commonly associated with rice production (methane emissions, surface water eutrophication, heavy metal accumulation in rice grains).

## Materials and methods

### Plant material and growth conditions

A total of 22 commercial rice varieties/experimental lines (Source data 1) were screened for low methane emissions under greenhouse conditions. The phytotron settings were 14 h light/10 h dark at 30 °C/21 °C, with constant relative humidity of 80% and light intensity of 400 μmol photons m^−2^·s^−1^. For the field trial in central Sweden (Uppsala), the low methane emission cultivar “Heijing 5” was selected as a test line and “Nipponbare” (Nipp) as a control (both japonica rice varieties). Because growth and development of the Nipp control line did not match that of Heijing 5 plants at the Uppsala site and Nipp plants did not reach the flowering stage, stands of *Acorus calamus* L. growing in nearby wetland were checked for methane emissions. In all experiments, rice seeds were germinated for 4 days on water-submerged filter paper in darkness at 28 °C and then transferred to the soil as described by Fei et al. (2020). The plants used in the field trials were grown for 10 days in a greenhouse before being transplanted to an open field at 25-cm plant spacing, replacing the natural wetland vegetation with rice. The open field site was located close to the Fyris river in Uppsala, Sweden (59.81° N, 17.67° E). According to World Weather Online (https://www.worldweatheronline.com), average night/day temperature at Uppsala ranged from 12 to 22 °C during the cultivation period (June–August 2020). Irrigation was performed using water from the Fyris river, which complies with the regulations on agricultural water quality (https://www.fao.org/3/ t0234e/t0234e00.htm#TOC). Through controlling the pumping time, the rice plants were covered with 5 cm of water on average. A total of 4000 Heijing 5 seedlings were planted evenly in four tents, while 12 Nipponbare (Nipp) seedlings were planted in a separate tent as controls. In addition, 12 Heijing 5 seedlings and 12 Nipp seedlings were cultivated in open paddy without tent coverage. The tents were closed when the temperature was below 10 °C. During the growing season, the average night/day temperature in the tent ranged from 15 to 32 °C, based on thermometer readings in each tent.

During preliminary and test screening in the phytotron, the rice plants were grown in cylinder-type pots (30 cm high, rim diameter 29 cm, base diameter 19 cm) with organic soil containing plant residues. For comparison, separate field trials were performed in Nanjing, China (32.05° N, 118.77° E), under temperatures ranging from ~ 22 to 35 °C and 80% humidity, in spring 2021 (July–September), essentially as described by Su et al. (2015). In the Nanjing field trials, the performance of Heijing 5 was compared with that of the elite rice variety Suxiangjing, which is a local rice variety in Jiangsu, China. Phenotypic analysis of the rice plants was performed as described by Fei et al. (2020).

### Collection and measurement of methane emissions

**Fig. 1:**
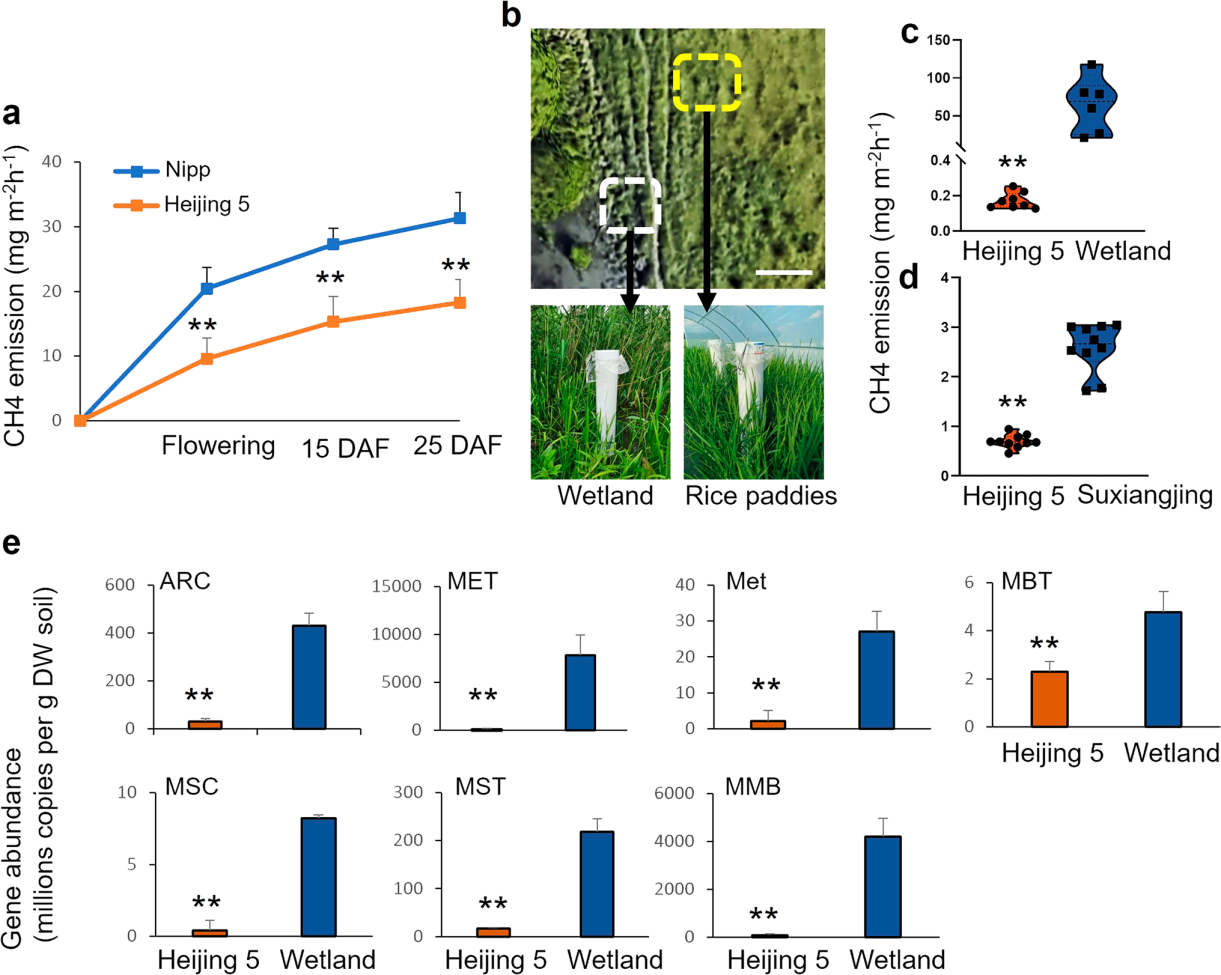
Characterisation of methane emissions. Shown in **a** are the levels of methane emission from phytotron grown Heijing 5 and Nipp plants at different developmental stages. The pictures in **b** show the set-up for methane collection in the Uppsala field trial. The study area was zoomed a geo-referenced from Google Earth. Sampled areas of wetland and rice paddies in Uppsala are marked with white dotted square and yellow dotted square, respectively. Shown in **c** and **d** are the levels of methane emissions from the field trials in Uppsala and Nanjing, respectively. Shown in **e** is the abundance of methanogenic communities estimated using qRT-PCR. Samples were taken from the rhizospheric part of Heijing 5 in the paddies and from rhizospheric soil from *Acorus calamus* L. plants as a control. Quantification was performed for total archaea (ARC) and methanogens (MET) and the orders Methanosaetaceae (MST), Methanosarcinaceae (MSC), Methanobacteriales (MBT), Methanomicrobiales (MMB), and Methanocella-specific (Met). DAF, day after flowering. GraphPad was used in **c** and **d**. Asterisks indicate a statistical differences of methane emission between Heijing 5 and Nipp in **a** (*n*=3), between Heijing 5 and Suxiangjing in **d** (*n*=10), and rice paddies compared to wetlands in **c** (*n*=8) and **e** (*n*=3), significant at *p* ≤ 0.01 (**) (Student’s *t*-test). Bar=5 m in **b**

Methane was collected as described previously (Su et al. 2015). In brief, methane was collected from flowering plants by covering the individual rice plant with a sealed plastic cylinder (diameter 15 cm, height 95 cm) for 15 min (Fig. 1b), starting at 14.00 h. Gas samples (2 × 50 mL) were taken from the headspace of each plastic cylinder using a syringe and pooled in a sealed vial. In the initial screening for low methane emission cultivars, methane emissions were measured at the flowering stage. In later tests, methane emissions from Nipp and Heijing 5 plants (*n*=3) growing in a climate-controlled phytotron were measured on the day of flowering and at 15 days after flowering (DAF) and 25 DAF. In the Uppsala field trials, emissions from eight Heijing 5 plants (*n*=8) were measured at flowering stage. On the same occasion, eight individual flowering plants of the wild grass *Acorus calamus* L. growing in surrounding wetland were randomly selected and checked for methane emissions. In the Nanjing control fields, methane emissions were measured on 10 plants when the Heijing 5 plants had reached the flowering stage. All samples were analysed by gas chromatography with appropriate methane standards. An air methane concentration of 1.8 ppm was used as the background for calculations. Methane flux was calculated as described by Su et al. (2015).

### qPCR quantification of methanogenic communities

Rhizosphere soil samples of Heijing 5 plants growing in tents and *Acorus calamus* L. plants growing in the surrounding wetland were collected from three different random plants. Collection started at 14.00 h, and each sample was taken from 5 cm depth. Total genomic DNA was extracted from 500 mg of fresh rhizosphere soil according to protocols described in the FastDNA Spin Kit for soil (MP Biomedicals LLC, USA). Abundance of methane-related microbes was quantified by quantitative real-time PCR (qPCR) using methanogen group-specific primers (Table S1) and the qPCR programme’s listen-in (Table S2). The abbreviations of each methanogenic group are as follows: MST (Methanosaetaceae), MSC (Methanosarcinaceae), MBT (Methanobacteriales), MMB (Methanomicrobiales), ARC (archaea) and MET (methanogens), and Met (*Methanocella*-specific) (Narihiro et al. 2011). Students’ *t*-test was used to analyse for statistical significance.

### Determination of total macronutrient (N, P, K) and heavy metals (As, Cd, Pb)

Water samples collected before entering the field (inflow) and flowing back into the Fyris (outflow) (both *n*=4) were analysed for total N, P, and K. Rice paddy soil (1–10 cm deep) and mature raw rice grains were analysed for total As, Cd, and Pb (*n*= at least 4). All chemical analyses on water, soil, and mature grain samples were performed at the accredited laboratory (No. ISO/IEC 17025: 2005 SWEDAC 1125) at Eurofins Sweden, using protocols detailed in Fei et al. (2020).

### RNA extraction and RNA-seq analysis

Leaves for RNA extraction were collected at the later tillering stage, starting at 14.00 h. Four replicates were used (*n*=4), and leaves from three plants in each replicate batch were ground into powder in liquid nitrogen. Approximately 30 mg powder was used for total RNA isolation, using the Spectrum Plant Total RNA Kit (Sigma-Aldrich) according to the manufacturer’s protocol. Isolated RNA was stored at −70 °C for further RNA sequencing experiments. In brief, samples with RNA Integrity Number (RIN) value ≥ 7 were used to synthesise mRNA libraries using the TruSeq stranded mRNA library preparation kit (Illumina, USA). The resulting libraries were sequenced with paired-end 150 bp reads using the NovaSeq 6000 platform at SciLifeLab in Stockholm (NBIS, Sweden), resulting in approximately 15 M read pairs per sample. Four biological replicates were used in each sequencing experiment. The original sequencing data were defined as raw reads. Clean reads were generated from the raw reads after removing low-quality reads, mismatches, and adaptor sequences. All downstream analyses were based on clean data.

The read data were mapped to *Oryza sativa* IRGSP-1.0 in Gramene via Hisat2. Differential expression analysis was performed using DESeq2 R package (Love et al*.* 2014). The resulting *p*-values were adjusted using the Benjamini-Hochberg approach for checking false discovery rate. Genes with significant threshold padj value < 0.05 and fold change > 2 were considered to be differentially expressed. Gene annotations were performed and determined using the reference genome from Gramene (https://www.gramene.org/). Venn diagrams were created on the website (https://bioinformatics.psb.ugent.be/webtools/Venn/). Panther (http://www.pantherdb. org/) was used to perform Gene Ontology (GO) analysis with Fisher statistic. VolcaNoseR (https://huygens.science.uva.nl/VolcaNoseR) was used for volcano plot analysis.

### Experimental design

The design of the experiments is shown in Table S3.

### Statistical analysis

To test for significant differences, measured methane emissions, micronutrient concentrations, and qPCR data were analysed using Students’ *t*-test, with differences considered significant differences at *p*< 0.05. For phenotypic analysis, 30 plants from each of four tents in the Uppsala field and eight individual plants in the Nanjing field were selected randomly.

## Results

### Screening for low-methane rice varieties

To identify a possible naturally occurring low-methane rice variety, 22 rice varieties and experimental lines were screened for methane emissions using gas chromatography (Source data 1). Emission rates from the different lines screened ranged from 3.5 to 24.5 mg·m^−2^·h^−1^ (Fig. S1). The variety with the lowest emission rates was the local variety Heijing 5, from Heilongjiang province, China. Methane emissions from the Heijing 5 rice plants under greenhouse conditions ranged from 9.60 to 18.27 mg·m^−2^·h^−1^ and were approximately 50% lower than those from the Nipp control plants (20.42–31.3 mg·m^−2^·h^−1^). Thus, the greenhouse experiments confirmed that Heijing 5 is a potential low-methane rice variety (Fig. 1a).

At the onset of flowering, methane emissions from Heijing 5 plants grown under field conditions at the Uppsala site were 0.17 mg·m^−2^·h^−1^ (Fig. 1b, c). Nipp plants did not reach the flowering stage (Fig. S2), but methane emissions from *Acorus calamus* L. plants growing in adjacent wetland were 64 mg·m^−2^·h^−1^, which was significantly higher than the emissions rate for Heijing 5 plants (Fig. 1b, c). At the Nanjing field site, methane emissions from Heijing 5 cultivation were 0.70 mg·m^−2^·h^−1^, while the local elite rice variety Suxiangjing showed fourfold higher methane emission rates (2.59 mg·m^−2^·h^−1^) (Fig. 1d). Hence, the initial screening and subsequent tests confirmed that Heijing 5 is a low methane emissions rice variety and that cultivation of Heijing 5 is associated with low methane emissions.

To determine whether the difference in methane emissions between Heijing 5 and *Acorus calamus* L. plants was reflected in the abundance and composition of soil methanogenic communities, the copy numbers of different methanogens in rhizosphere samples collected at flowering time were compared using qRT-PCR (Fig. 1e). In line with the observed methane emissions, the copy numbers of MST, MSC, MBT, MMB, ARC, Met, and MET were all significantly lower in the rice rhizosphere samples than in the *Acorus calamus* L. rhizosphere samples. MMB was the dominant methanogen in both rice paddy and wetland rhizosphere samples (Fig. 1e).

### Cultivation of paddy rice in Sweden

**Fig. 2:**
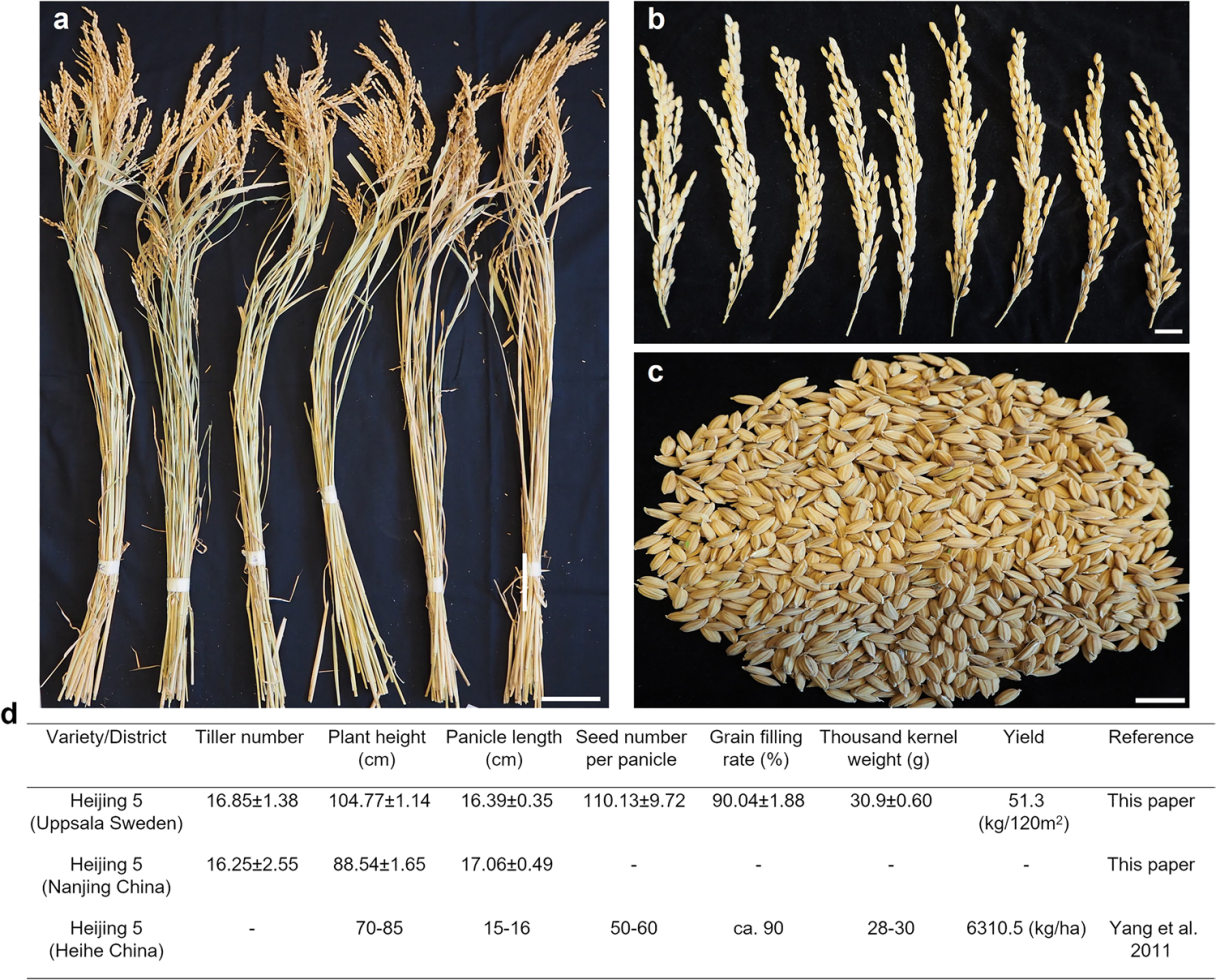
Phenotypic analysis of Heijing 5. The pictures show whole Heijing 5 plants in **a** a close up of panicles in **b** and a display of Heijing 5 grains in **c**. Shown in **d** is the statistical analysis of yield-related phenotypes. Heijing 5 (*n*=120), cultivated in Nanjing, China (*n*=8), and Heihe, China, was used as control. g, gramme; kg, kilogramme; ha, hectares. Bar=10 cm in **a** and 2 cm in **b** and **c**

Apart from studying methane emissions, the field trials in Uppsala examined whether Heijing 5 could complete an entire growth period in the relatively short, cold summers at high latitude. Based on the appearance of whole rice plants, panicle length, and grain shape (Fig. 2), growth of Heijing 5 was very uniform with respect to plant height and number of tillers (Fig. 2a). Prominent characteristics of Heijing 5 were that more than 90% of the grains were fully filled (Fig. 2b) and grain shape was regular (Fig. 2c). Comparison of Heijing 5 plants grown in Uppsala with plants grown in Heihe, China (50.15° N; Yang et al. 2011), showed that the Uppsala plants grew taller and had a greater number of seeds than the Heihe plants but had similar panicle length, grain filling rate, and thousand kernel weight (Fig. 2d). The Heijing 5 plants grown in Uppsala also had similar numbers of tillers and panicle length as plants grown in Nanjing (32.05° N) (Fig. 2d). Thus, the results indicated that the performance of Heijing 5 cultivated in Sweden and at two sites in China was similar in terms of growth and yield potential.

**Fig. 3:**
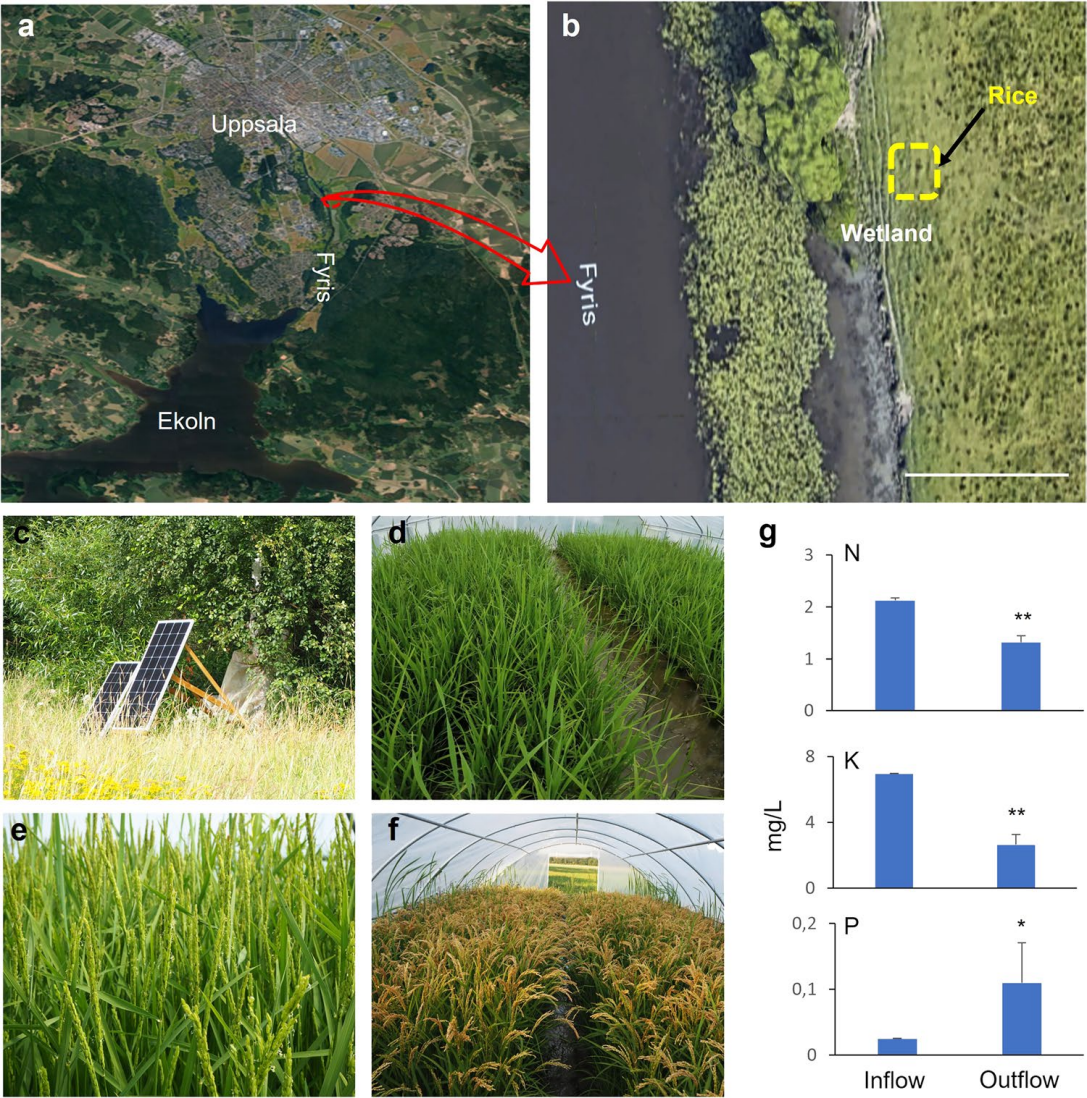
Location of the field trial in Uppsala. The pictures shows the location of Heijing 5 cultivation in **a** and the river Fyris from which water and nutrient for the cultivation was obtained in **b**. A dotted square marks the rice cultivation area. The study area was zoomed and geo-referenced from Google Earth. As shown in **c**, solar power was used as the energy source to pump the water from river Fyris. Pictures **d**–**f** show Heijing 5 at the tillering (**d**), flowering (**e**), and ripen stage (**f**) The graphs in **g** shows the nutrient levels in the water that flow into the rice paddies (inflow) and the water that flow back into the river (outflow). Asterisks indicate a statistical difference of outflow compared to the inflow with 4 replicates (*n*=4), significant at *p* ≤ 0.05 (*) or *p* ≤ 0.01 (**) (Student’s *t*-test). N, nitrogen; K, potassium; P, phosphorus. Bar=30 m in **b**

Water from the Fyris river at the Uppsala site (inflow) was analysed for suitability for irrigation purposes, while drainage water re-entering the river (outflow) was analysed to assess how rice cultivation affected the nutrient load to the river. The annual nutrient loads entering the river Fyris are reported to be 120,323 tonnes N and 3463 tonnes P (Piniewski et al. 2021). These loads, which originate from the city of Uppsala and upstream farmland, lead to enrichment of river water with nutrients and eutrophication. To test whether rice cultivation around the river could absorb some of the nutrient load in river water and whether this could sustain normal Heijing 5 growth without additional fertilisation, Heijing 5 rice was grown on an area of 120 m^2^ close to the river (Fig. 3a, b). Solar power was used to pump water from the river, to save energy (Fig. 3c). On assessing rice developmental stages (i.e. tillering, flowering, and full grain filling), it was concluded that the nutrient levels in river water were sufficient to sustain rice growth, without any additional fertiliser needed (Fig. 3d–f). Inflow water contained 2.2 mg N·L^−1^ and 7 mg K·L^−1^, while outflow water only contained 1.2 mg N·L^−1^ and 3 mg K·L^−1^ (Fig. 3g), which corresponded to a 45% reduction in N and 57% reduction in K. This suggests that the rice paddies assimilated N and K, lowering the load to the river. However, the P concentration was higher in outflow than in inflow water (Fig. 3g).

### Low heavy metals in Heijing 5 grain and paddy soil

Since soils in Sweden generally contain relatively low levels of heavy metals such as As, Pb, and Cd, concentrations of those heavy metals in rice grains from Heijing 5 plants grown in Uppsala were expected to be low. The measured As, Pb, and Cd concentrations in Heijing 5 grain were consistently lower than literature values (As, 0.064 ± 0.011 mg·kg^−1^; Pb, < 0.02 mg·kg^−1^; Cd, 0.055 ± 0.025 mg·kg^−1^) (Table 1). They were also much lower than the international thresholds for foods (As, 0.35 mg·kg^−1^; Pb, 0.2 mg·kg^−1^; Cd, 0.4 mg·kg^−1^) set by FAO and WHO (CXS 193-1995). The low concentrations of heavy metals in Heijing 5 grain reflected the low concentrations in Uppsala paddy soil (As, 6.0 ± 0.51 mg·kg^−1^; Pb, 20.75 ± 1.89 mg·kg^−1^; Cd, 0.94 ± 0.83 mg·kg^−1^) compared with paddy soil in other countries (Table 2).

**Table 1:** Heavy metal concentrations in rice grains from different sites worldwide

Country	Total arsenic Mean (mg·kg^−1^)	Total lead Mean (mg·kg^−1^)	Total cadmium Mean (mg·kg^−1^)	Reference
Uppsala, Sweden	**0.064 ± 0.011**	**< 0.02**	**0.055 ± 0.025**	**This paper**
Bangladesh	0.137	0.375	0.088	Williams et al. (2005, 2006), Meharg et al. (2009), Rahman et al. (2014a, 2014b), Ahmed et al. (2015)
India	0.066	0.267		Williams et al. (2005, 2006), Meharg et al. (2009), Rahman et al. (2014a, 2014b)
China	0.187	0.28 ± 0.06	0.229	Meharg et al. (2009), Zhu et al. (2008), Fei et al. (2020), Liu et al. (2016), Shi et al. (2020)
Thailand	0.137	0.419	0.329	Williams et al. (2006), Meharg et al. (2009), Rahman et al. (2014a, 2014b), Suwatvitayakorn et al. (2020)
Philippines	0.07	-	-	Williams et al. (2006)
Japan	0.19	-	-	Meharg et al. (2009)
Pakistan	0.082	0.067	-	Rahman et al. (2014a, 2014b)
Vietnam	-	0.016	-	-
Australia	0.27	0.375	-	Rahman et al. (2014a, 2014b)
Italy	0.275	0.317	0.034 ± 0.007	Rahman et al. (2014a, 2014b), Imperato et al. (2003), Zoli et al. (2021)
Spain	0.23	-	-	Torres-Escribano et al. (2008), Meharg et al. (2009), Tattibayeva et al. (2016)
France	0.28	-	-	Meharg et al. (2009)
Portugal	0.36	-	-	Tattibayeva et al. (2016)
FAO	0.35	0.2	0.4	CXS 193-1955

**Table 2:** Heavy metal concentrations in soil at different sites worldwide

Country	Total arsenic Mean (mg·kg^−1^)	Total lead Mean (mg·kg^−1^)	Total cadmium Mean (mg·kg^−1^)	Reference
Uppsala, Sweden	**6.0 ± 0.51**	**20.75 ± 1.89**	**0.94 ± 0.83**	**This paper**
Bangladesh	11.41–61.54	25.9	-	Hossain et al. (2008), Ahsan et al. (2009)
India	37.7	38.1	1.73 ± 0.36	Shrivastava et al. (2017), Singh et al. (2014), Yadav et al. (2017), Sharma et al. (2018)
China	2.5–19.2	24.07 ± 4.54	1.0	Jiang et al. (2014), Liu et al. (2016), Chen et al. (2018)
Sweden	2.8 ± 0.1	-	-	Fei et al. (2020)

### Molecular analysis of the adaption mechanism of Heijing 5 to high latitude

Despite the higher latitude at the Uppsala site and the lower average daily temperatures (range 12–22 °C during the growing period), Heijing 5 plants grown in Uppsala followed a similar growth curve as those grown at the sites in China (Fig. 3d–f). This indicates that Heijing 5 is adapted to growth at high latitudes and possibly also lower temperatures. In contrast, the control rice line Nipp did not reach the flowering stage under the conditions prevailing at the field site in Uppsala. To assess whether this adaptation was reflected in differences at the molecular level between Heijing 5 and Nipp, massively parallel mRNA sequencing from leaf samples collected at the later tillering stage of both cultivars was performed.

To test the effect of temperature and day length on rice growth, samples of Heijing 5 and Nipp were divided into two groups: (i) plants grown in open-air field trials (Heij-O, Nipp-O) and (ii) plants grown in protective tents (Heij-T, Nipp-T). Light intensity and day length were similar in both experimental set-ups, so commonly differentially expressed genes (DEGs) in Heij-O vs Nipp-O and Heij-T vs Nipp-T may represent genes responding to the high-latitude day length conditions in Uppsala, with DEGs exclusive to open-air plants more likely to be involved in temperature adaptation or acclimatisation.

**Fig. 4:**
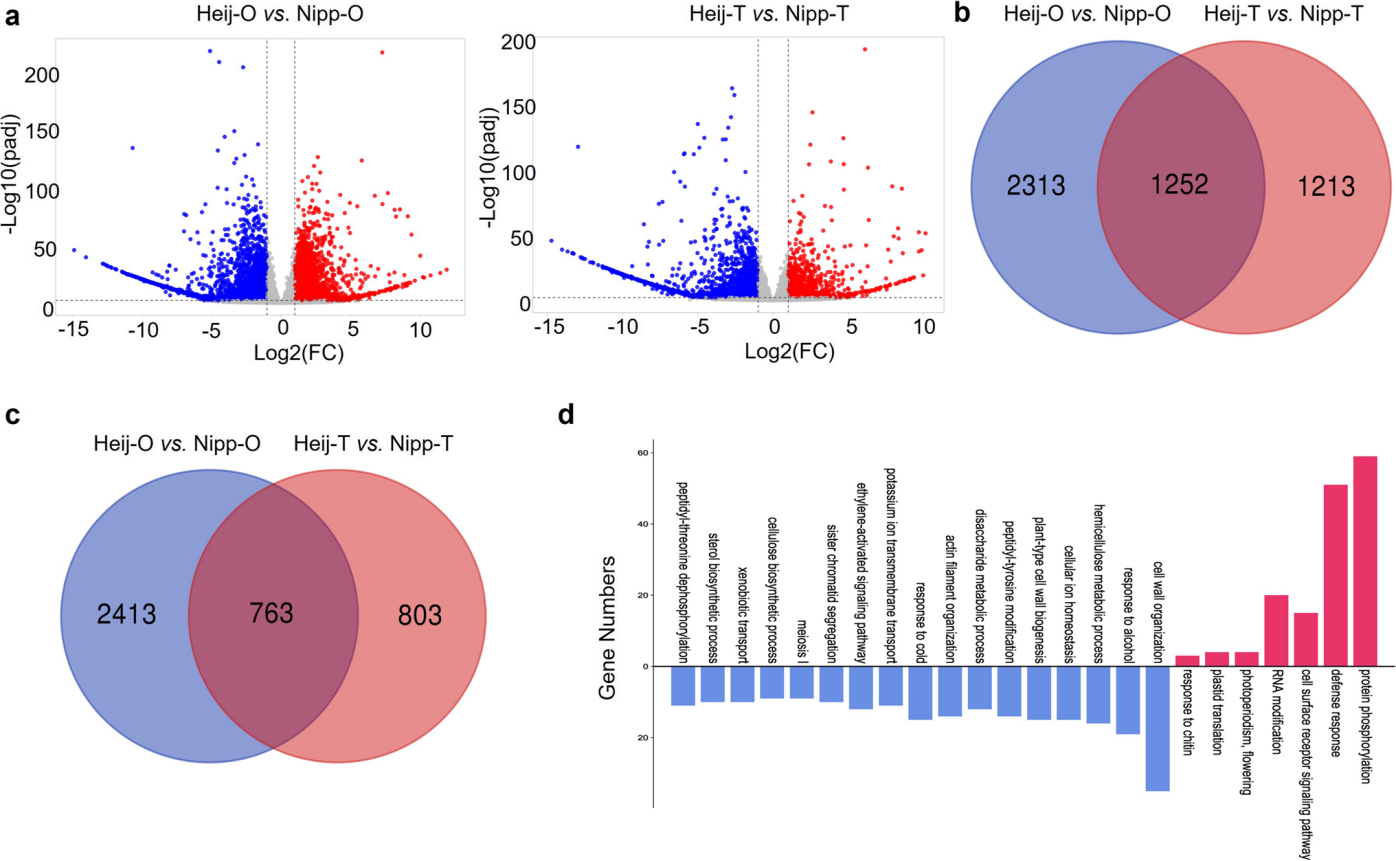
Transcriptomic analysis of Heijing 5 in response to high-latitude growth conditions. Shown in **a** are volcano plots representing differentially expressed genes (DEGs) according to fold change cut of > 2 and adjusted *p*-value of < 0.05. Comparisons were made on rice plants grown in tents (Heij-T vs Nipp-T) and without tent (Heij-O vs Nipp-O). Red dots and blue dots represent significantly up- or down-regulated genes, respectively; grey dots indicate genes that were not significantly differentially expressed. Venn diagrams show the number of down-regulated DEGs (**b**) and up-regulated DEGs (**c**) that overlap between the comparisons (Heij-T vs Nipp-T and Heij-O vs Nipp-O). The graph in **d** shows the output from the Gene Ontology (GO) analysis based on biological process categories. The blue colour indicates that the categories are exclusive Heij-O vs Nipp-O, whereas red colour indicates that the categories are common for both comparisons. Log2 fold change > 1 and padj < 0.05 were set up for DEG and GO enrichment. Four replicates (*n*=4) were used. FC, fold change; Heij-O, field cultivation of Heijing 5 without tent; Nipp-O, field cultivation of Nipponbare without tent; Heij-T, field cultivation of Heijing 5 with tent; Nipp-T, field cultivation of Nipponbare with tent

To identify differentially expressed genes, we used a combined cutoff of fold change > 2 and adjusted *p*-value < 0.05, here represented by volcano plots (Fig. 4a). On comparing Heij-O and Nipp-O plants, we identified 6741 DEGs, of which 3176 were significantly up-regulated and 3565 were significantly down-regulated (Source data 2). In the samples collected from plants grown in experimental tents (Heij-T and Nipp-T), we identified 4031 DEGs, of which 1566 were significantly up-regulated and 2465 were down-regulated (Source data 3). Based on the number of DEGs in the two comparisons, more genes from Heijing 5 were differentially expressed in plants grown in the open air. Among these, 2313 genes were exclusively down-regulated, and 2413 were only up-regulated, in Heij-O plants (Fig. 4b, c). Gene Ontology analysis of the 2313 down-regulated genes identified seventeen GO terms as being significantly enriched (Fig. 4d). Among these, the GO term “response to cold” (GO:0009409) included 15 genes up-regulated in Nipp-O plants compared with Heij-O plants (Table S4a), supporting the suggestion that Heij-O is cold-acclimatised. Fifteen GO terms were significantly enriched among the up-regulated genes in the Heij-O vs Nipp-O comparison (Fig. S3), but none of these terms had an obvious connection to cold acclimatisation. As a complement, we performed a directed search for genes involved in cold tolerance among the genes up-regulated only in Heij-O plants. This search identified six genes that encode proteins with putative roles in cold tolerance (Table S4b).

Next, we analysed the composition of differentially expressed genes that were common for both comparisons, i.e. Heij-O vs Nipp-O and Heij-T vs Nipp-T. In this comparison, we identified 763 genes that were commonly up-regulated, whereas 1252 were commonly down-regulated (Fig. 4b, c). We considered the up-regulated genes to be most likely to be involved in the photoperiod response. GO enrichment analysis of the 763 up-regulated genes identified seven GO terms as being significantly enriched (FDR < 0.05). Among these, the GO term “photoperiod and flowering” (GO:0048573) included four genes (Table S5), of which two encoded members of the PEBP gene family (*RF1* and *Hd3a*). Notably, the proteins encoded by *RF1* and *Hd3a* are believed to constitute the mobile signal florigen in rice and to regulate rice flowering under short-day conditions (Komiya et al*.* 2008).

## Discussion

### The Heijing 5 variety has potential for high-latitude cultivation

At present, rice is commonly cultivated up to latitude 50.15° N (Yang et al., 2011), but our pilot study indicated that Heijing 5 could be cultivated successfully in Uppsala, at latitude 59.8° N, suggesting that it is possible to expand the rice cultivation area northwards. We confirmed this in a field trial in Uppsala, Sweden, where Heijing 5 plants produced large numbers of grains per panicle and displayed high rates of complete grain filling (Fig. 2), suggesting considerable yield potential even at high-latitude locations. The estimated yield was 4275 kg·ha^−1^, based on the yield of 51.3 kg and plant density of 4000 in the 120 m^2^ trial area. In comparison, Heijing 5 cultivated in Heihe, China, yielded 6310.5 kg·ha^−1^ with a planting density of 5.25–6 million plants per hectare. The lower-density planting explains the relatively low yield of Heijing 5 rice plants in Uppsala, because there is a positive correlation between rice yield and planting density (Liu et al. 2019).

This study focused on the growth characteristics of Heijing 5 plants and associated methane emissions. Of course, introducing rice as a new crop in high-latitude countries would require at least partial transformation of the farming landscape, which could impact the environment through, e.g. increased water usage and nutrient leaching. In Sweden, farming currently accounts for approximately 3% of the total volume of water usage (SCB, 2020), and 72% of the water used in agriculture is for irrigation. If rice cultivation was to be implemented, that percentage would likely increase, affecting water availability. However, some farmland close to lakes and riverbeds that is now protected from inundation by embankments (https://webbutiken.jordbruksverket.se/sv/artiklar/%20klimatforandringarna-och-invallningen.html) could potentially be used for rice cultivation without any serious effects on water quality or availability. The present study used water from the Fyris river, which passes through rural farmland and the city of Uppsala and is relatively rich in nutrients, and was able to sustain normal growth of the rice plants. Therefore, no extra fertiliser was needed in the field trial, in contrast to ordinary rice cultivations. Hence, the field trial did not add to the total amount of nutrients in the environment and in fact the rice plants appeared to use some of the N and K in the water to sustain growth. However, some nutrients may have been lost from the paddy soil by percolation to the groundwater. The concentration of P was higher in outflow water than in inflow water, possibly because of increased loss of P stored in the rice paddy soil. Therefore, before any large-scale implementation of rice cultivation, the results in this field trial must be confirmed in studies of soil composition and of flows of nutrients to groundwater and surface waters. Moreover, irrigation water quality should be further analysed to ensure that it does not add residues of household chemicals or pharmaceuticals to the rice fields. In an international comparison, the levels of heavy metals in grain of Heijing 5 plants grown in Uppsala were relatively low, which can be attributed to the unpolluted soils in Sweden. However, close monitoring of water and soil quality is important so that any new farming activity does not contribute to accumulation of heavy metals and soil deterioration.

### Heijing 5 is a low methane emission variety

Apart from enabling expansion of the putative cultivation area of rice into high-latitude countries, the Heijing 5 variety has the environmental benefit of being associated with reduced methane emissions. This was observed under both climate-controlled greenhouse conditions and field conditions in Sweden and China in this study. Since Nipp did not initiate flowering under field conditions in Uppsala, a comparison could not be made at that stage of development, so an adjacent local plant, e.g. *Acorus calamus* L., was used as reference. The results were consistent with the previous findings in previous studies comparing methane emissions from wild grasses and commercial rice cultivars indicating that natural wetlands often are associated with higher methane emission rates than cultivated rice paddies (Oda et al. 2018, 2019). One explanation for the lower methane emissions from rice cultivars relative to wild grasses could be that crops are generally selected for high yield and therefore allocate more carbon to aboveground tissues. Allocation of carbon from root to shoot may reduce the amount of organic carbon available for methanogenesis (Liu et al. 2017). We observed much greater variation in methane emissions from *Acorus calamus* L. plants than from Heijing 5 plants, although average emissions rates were consistently higher in the former. The *Acorus calamus* L. plants used in the study were located at different spots in the same area as the cultivated Hejing5 plants, and differences in soil composition probably contributed to the variation in methane emission rates for *Acorus calamus* L. plants. The methane emissions from Heijing 5 plants varied in the three experiments, with the highest emissions occurring under controlled greenhouse conditions (9.60 mg·m^−2^·h^−1^) and considerably lower emissions in the field trials (0.70 mg·m^−2^·h^−1^ and 0.17 mg·m^−2^·h^−1^ in China and Sweden, respectively). One possible reason for the difference in the methane emission rates between the experiments could be differences in cultivation temperature, since it has been shown that changes in temperature influence methane production by shifting or reducing microbial activities (Peng et al. 2008). Thus, the higher emissions in the phytotron were caused by stable and relatively high temperatures (30/21 °C day/night temperatures), an ideal temperature range for methanogens, while the lower temperatures in the Chinese and Swedish field trials, especially at night, contributed to lower methane emissions by the same mechanism. Methanomicrobiales (MMB) and Methanosaetaceae (MST), known as hydrogenotrophic and acetoclastic methanogens, respectively (Alpana et al*.* 2017), were predominant in both rice paddy and wetland soils, which indicates that hydrogen and acetate were both abundant in the Uppsala rice paddy.

### Cold-resistant and early-flowering genes participate in adaption of Heijing 5 to high-latitude sites

To study the genetic mechanism underlying the adaption of Heijing 5 to high-latitude regions, we performed transcriptome analysis using massively parallel sequencing of mRNA samples derived from leaf samples of Heijing 5 and Nipp plants grown in open fields or under tents to protect the plants from cold night temperatures. This set-up allowed us to distinguish genes that were part of adaptation to high-latitude day length and candidate genes involved in cold acclimatisation.

Nipp plants grown without a protective tent expressed several genes connected to the GO term “response to cold” (GO:0009409). The up-regulated genes in this GO category encoded, e.g. three members of the SYG1/Pho81/XPR1 (SPX) gene family, which have also been connected to P starvation (Wang et al. 2013; Liu et al. 2018). The discovery of a “response to cold” GO item in Nipp, but not in Heijing 5, indicates that Nipp is more sensitive to cold stress than Heijing 5. In this GO category, we also identified genes encoding MYB and NAC domain-containing transcription factors shown to respond to cold treatments (Yang et al. 2012; Dong et al. 2021). Heijing 5 and Nipp are japonica rice cultivars which typically induce flowering in response to long days (Izawa et al. 2009). In rice, the CONSTANCE ortholog HEADING DATE1 (HD1) suppresses flowering under long-day conditions (Yano et al*.* 2000). In contrast, the B-type response regulator EARLY HEADING DATE1 (EHD1) activates flowering independent of *HD1* (Doi et al. 2004). *HD1* and *EHD1* influence flowering time by simultaneously regulating the florigen encoding gene *EHD3A* at the transcriptional level. Furthermore, it has been suggested that natural variations in the balance between *HD1* suppression and *EHD1* promotion activities have contributed to the domestication of rice cultivars adapted to different day length conditions (Yano et al. 2000). Interestingly, we identified elevated transcript levels of *EHD3A*, and its closest paralog *RFT1*, in Heijing 5 plants compared with Nipp plants. This suggests that Heijing 5 is an early-flowering cultivar that responds to successive increases in day length by activating *EHD3A* transcription earlier in the growing season than the Nipp cultivar. It is also tempting to speculate that differences in the activities of *HD1* and *EHD1*, and the subsequent transcriptional activation of *EHD3A*, contribute to the adaptation of Heijing 5 to the high-latitude growing conditions present in central Sweden.

## Conclusions

This study identified a putative low methane emissions rice cultivar (Heijing 5) that can potentially be grown under high-latitude growing conditions. In combined field and pot experiments, cultivation of Heijing 5 was shown to be associated with reduced methane emissions compared with high-yielding rice cultivars and wild grass (*Acorus calamus* L.) plants growing adjacent to the field plots. Heijing 5 plants grown under high-latitude conditions in Uppsala, Sweden, showed similar high yield potential to that reported for Heijing 5 grown in its province of origin (Heilongjiang) in China.

It can be concluded that Heijing 5 has good yield potential when cultivated under high-latitude conditions and that cultivation of Heijing 5 in Uppsala is not associated with the soil accumulation of heavy metals such as As, Pb, and Cd. Massively parallel sequencing of mRNA samples identified candidate genes involved in the adaptation of the Heijing 5 to growth at high latitudes, with transcriptional regulation of the flowering-inducing genes EHD3A and RFT1 appearing to be instrumental in this adaptation.

## Supplementary information

ESM 1

ESM 2

ESM 3

ESM 4

## Data Availability

All data generated or analysed during this study are included in this published article or its supplementary information. The materials are available on request from the corresponding author. Raw data from RNA sequencing will be deposited in a public database upon publication.
