# Supplementary material for: Characterisation of a low methane emission rice cultivar suitable for cultivation in high latitude light and temperature conditions
Source: Environ Sci Pollut Res Int. 2023 Jul 27;30(40):92950–62. doi: 10.1007/s11356-023-28985-w (PMC10447601; doi:10.1007/s11356-023-28985-w)
Supplement: Supplementary file 1 — ESM 1 [file 11356_2023_28985_MOESM1_ESM.pdf]

Source data 1. Source clarification of rice line/varieties

The authors clarify that rice varieties Heijing 5 and Nipponbare are stored in our group which have been reported by Fei et al., 2020 and Su et al., 2015. Other lines/varieties came from Prof. Mingzhou Zhang, Jiliang University, Hangzhou China; Prof. Dianxing Wu, Prof. Qingyao Shu and Prof. Jinsong Bao, from Zhejiang University, Hangzhou China. Part of the elite rice varieties were purchased from market. The details are listed as below.

Purpose	Line/Variety	Source (name + institude)
Low methane rice Screening	Heijing 5	Chuanxin Sun group, SLU
Low methane rice Screening	Jiaxian 08	Qingxiao Shu, Zhejiang University
Low methane rice Screening	Zhengdao 413	Elite rice from market
Low methane rice Screening	Nipp	Chuanxin Sun group, SLU
Low methane rice Screening	GM645	Jinsong Bao, Zhejiang University
Low methane rice Screening	Jiahe218	Qingxiao Shu, Zhejiang University
Low methane rice Screening	Xiushui110	Qingxiao Shu, Zhejiang University
Low methane rice Screening	Dangyujing10	Elite rice from market
Low methane rice Screening	Xidao	Qingxiao Shu, Zhejiang University
Low methane rice Screening	CYP71A	Qingxiao Shu, Zhejiang University
Low methane rice Screening	Wuchang	Elite rice from market
Low methane rice Screening	Jiahua	Elite rice from market
Low methane rice Screening	Shijijiaozi	Elite rice from market
Low methane rice Screening	J2	Qingxiao Shu, Zhejiang University
Low methane rice Screening	GM034	Jinsong Bao, Zhejiang University
Low methane rice Screening	GM077	Jinsong Bao, Zhejiang University
Low methane rice Screening	Dangyujing 8	Elite rice from market
Low methane rice Screening	ZF05	Dianxing Wu, Zhejiang University
Low methane rice Screening	ZF06	Dianxing Wu, Zhejiang University
Low methane rice Screening	ZF900	Dianxing Wu, Zhejiang University
Low methane rice Screening	Xiushui314	Elite rice from market
Low methane rice Screening	Huayu	Elite rice from market
