# Supplementary material for: Characterisation of a low methane emission rice cultivar suitable for cultivation in high latitude light and temperature conditions
Source: Environ Sci Pollut Res Int. 2023 Jul 27;30(40):92950–62. doi: 10.1007/s11356-023-28985-w (PMC10447601; doi:10.1007/s11356-023-28985-w)
Supplement: Supplementary file 4 — ESM 4 [file 11356_2023_28985_MOESM4_ESM.docx]

**Supplementary Figures**


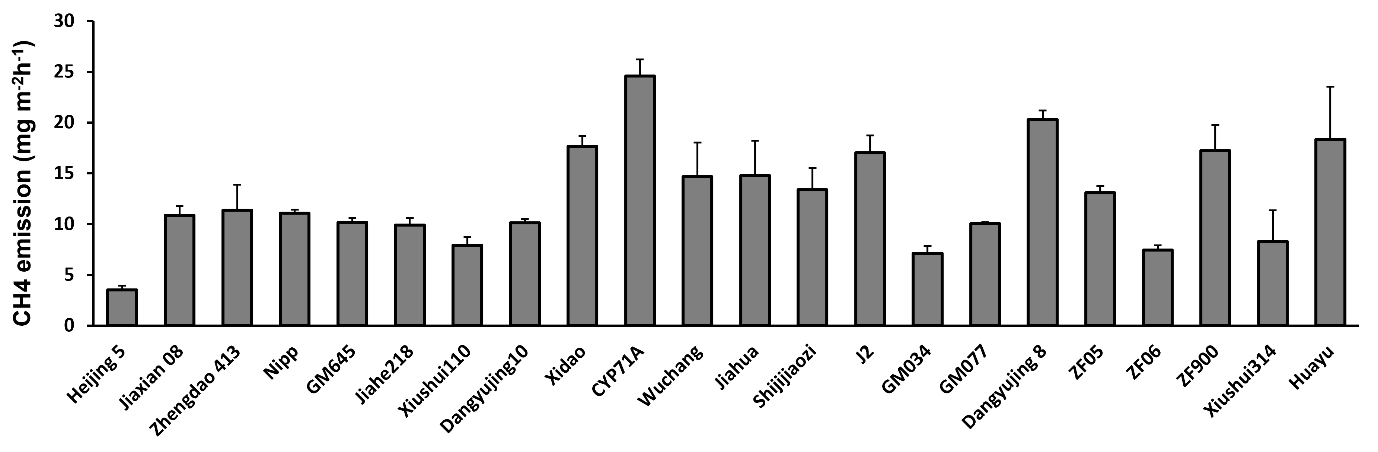
Fig. S1 Results of screening of different rice cultivars for low methane emissions (mean, n=3).


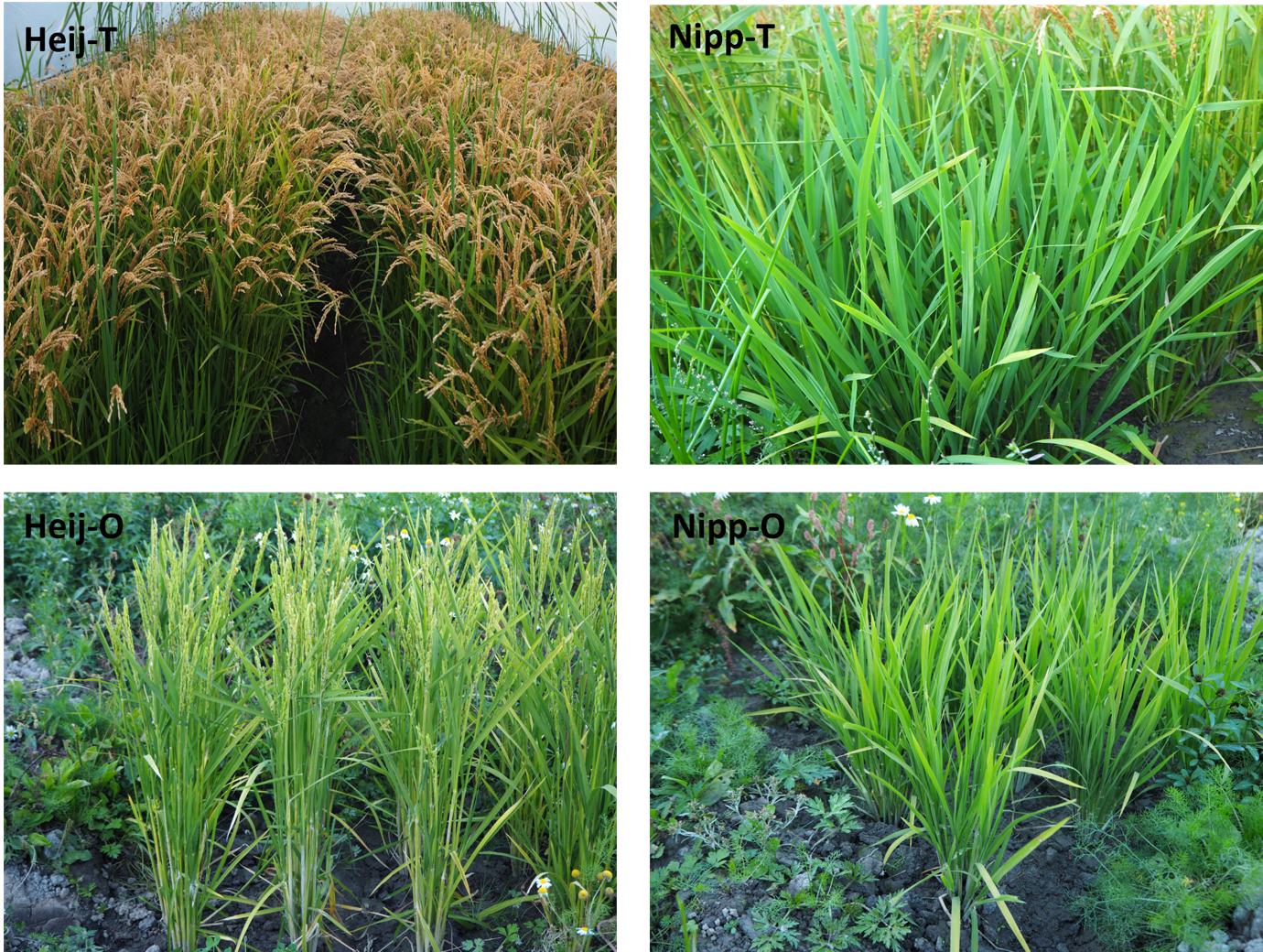


**Fig. S2** Comparison of growth status of Heijing 5 and Nipp plants grown in tents (Heij-T, Nipp-T) and in the open field (Heij-O, Nipp-O).

**
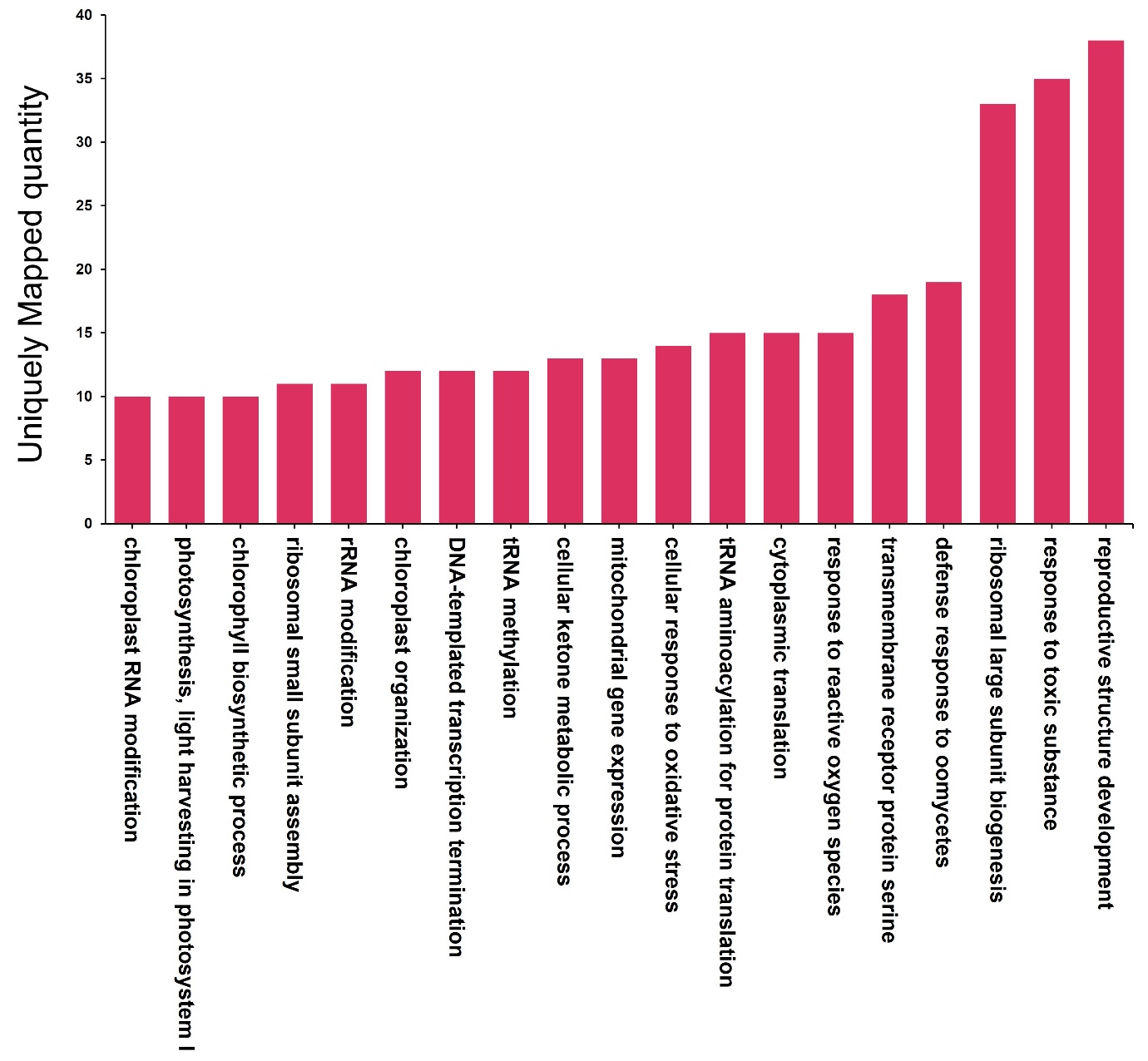
**

**Fig. S3** Gene ontology (GO) term enrichment from up-regulated genes in Heij-O *vs* Nipp-O, at padj <0.05 and gene number >10. Heij- and Nipp-O = cultivated in open field.

**Table S1** Oligonucleotides used in this work

| Primers used to quantify methanogenic groups | | |
| --- | --- | --- |
| Primer name | Gene name | Primer sequence (5' → 3') |
| MST702F | Methanosaetaceae (MST) | TAATCCTYGARGGACCACCA |
| MST862R | 16s RNA | CCTACGGCACCRACMAC |
| MET630F | Methanogen (MET) | GGATTAGATACCCSGGTAGT |
| MET803R | 16s RNA | GTTGARTCCAATTAAACCG |
| MSC380F | Methanosarcinaceae | GAAACCGYGATAAGGGGA |
| MSC828R | (MSC) 16s RNA | TAGCGARCATCGTTTACG |
| MBT857F | Methanobacteriales | CGWAGGGAAGCTGTTAAGT |
| MBT1196R | (MBT) 16s RNA | TACCGTCGTCCACTCCTT |
| MMB282F | Methanomicrobiales | ATCGRTACGGGTTGTGGG |
| MMB832R | (MMB) 16s RNA | CACCTAACGCRCATHGTTTAC |
| ARC787F | Archaea (ARC) 16s | ATTAGATACCCSBGTAGTCC |
| ARC1059R | RNA | GCCATGCACCWCCTCT |
| Met282F | Methanocella-specific | ATCMGTACGGGTTGTGGG |
| Met832R | (Met) 16s RNA | CACCTAGCGRGCATCGTTTAC |

**Table S2** qPCR programs used in this work

| Genes | Initial Denaturation | Denature | Anneal | Extend | Cycles |
| --- | --- | --- | --- | --- | --- |
| MST | 95°C for 7 min | 95°C for 40 s | 61°C for 1 min | 72°C for 40 s | 40 |
| MET | 95°C for 7 min | 95°C for 40 s | 60°C for 1 min | 72°C for 40 s | 40 |
| MSC | 95°C for 7 min | 95°C for 40 s | 60°C for 1 min | 72°C for 40 s | 40 |
| MBT | 95°C for 7 min | 95°C for 40 s | 58°C for 1 min | 72°C for 40 s | 40 |
| MMB | 95°C for 7 min | 95°C for 40 s | 66°C for 1 min | 72°C for 40 s | 40 |
| ARC | 95°C for 7 min | 95°C for 40 s | 60°C for 1 min | 72°C for 40 s | 40 |
| Met | 95°C for 7 min | 95°C for 40 s | 60°C for 1 min | 72°C for 40 s | 40 |

**Table S3** Experimental design in this work

|  | Methane emissions | Nutrition | Heavy metals | Yield | Transcriptome analysis |
| --- | --- | --- | --- | --- | --- |
| Uppsala (field trials) | Test | Test | Test | Test | Test |
| Pots (phytotron) | Test | N.A. | N.A. | N.A. | N.A. |
| China (field trails) | Test | N.A. | N.A. | Test | N.A. |
| Published data | N.Q: | Quote | Quote | Quote | N.Q. |

Test: experiment described in this paper. N.A.: Not Analysed, experiment not performed.

Quote: Referenced from published results. N.Q.: Not quote.

**Table S4** List of DEGs related to response to cold (GO:0009409) that were up-regulated in (a) Nipp-O and (b) (b) Heij5-O

(a)

| Gene ID | Log2 Foldchange | padj | Annotation |
| --- | --- | --- | --- |
| Os11g0453900 | -4,96847 | 0,000241 | Responsive to ABA gene 16D, (RAB16D) |
| Os10g0392600 | -3,37143 | 0,000988 | SPX domain-containing protein, negative regulation of phosphate signalling, Pi homeostasis |
| Os10g0389500 | -1,03164 | 8,05E-07 | Cold regulated protein 27 |
| Os09g0426800 | -1,6019 | 4,87E-13 | Homologue of WAX2/GL1, synthesis of leaf cuticular wax |
| Os06g0603600 | -1,29315 | 1,63E-11 | SPX domain-containing protein, phosphate (Pi) homeostasis, negative regulation of leaf inclination |
| Os04g0605500 | -1,43305 | 1,53E-15 | P-type IIB Ca(2+) ATPase, stress tolerance |
| Os04g0540900 | -2,97351 | 0,014407 | Receptor-like cytoplasmic kinase, salt tolerance, oxidative stress tolerance |
| Os04g0517100 | -1,48495 | 0,000377 | SG2-type MYB transcription factor, cold tolerance, resistance to fungal and bacterial pathogens, panicle development |
| Os03g0406100 | -2,94084 | 0,000322 | SPX domain-containing protein, negative regulation of phosphate signalling, Pi homeostasis |
| Os03g0315400 | -1,06149 | 0,027158 | R2R3-type MYB transcription factor, "Salt, cold and dehydration tolerance" |
| Os02g0661100 | -2,42068 | 7,14E-19 | Trehalose-6-phosphate phosphatase, trehalose biosynthesis, chilling tolerance |
| Os02g0621300 | -2,40899 | 7,61E-21 | Homologous protein of CER1, very-long-chain (VLC) alkane biosynthesis, regulation of anther development and plastid differentiation |
| Os02g0255500 | -1,26861 | 9,47E-22 | ABA receptor, pyrabactin resistance-like (PYL) abscisic acid receptor family protein, cold and drought stress tolerance, ABA-mediated inhibition of seed germination |
| Os01g0884300 | -1,16462 | 1,21E-31 | NAC transcription factor, drought tolerance, response to abiotic and biotic stresses |
| Os01g0831200 | -1,14569 | 3,01E-33 | BAG family protein, control of innate immunity and broad-spectrum disease resistance |

(b)

| Gene ID | Log2 Foldchange | padj | Annotation |
| --- | --- | --- | --- |
| Os01g0369200 | 1,270956 | 0,001309 | Component of cullin-RING E3 ubiquitin ligase (CRL) complex, salt/drought stress response, cold stress response |
| Os03g0108600 | 1,076016 | 2,86E-22 | DEAD-box RNA helicase, early chloroplast development under cold stress, cold tolerance, defence responses against biotic and abiotic stresses |
| Os03g0656900 | 1,442067 | 9,28E-38 | Plastid RNA-binding protein, regulation of chloroplast RNA metabolism, early chloroplast development under cold stress |
| Os04g0475500 | 1,69969 | 9,42E-23 | Pentatricopeptide repeat (PPR) protein, early chloroplast development under cold stress |
| Os06g0658900 | 1,118032 | 5,75E-26 | Homogentisate phytyltransferase (HPT), tocopherol (vitamin E) biosynthesis, plant development, cold tolerance |
| Os12g0210300 | 2,211892 | 6,55E-51 | Zeta class glutathione transferase, cold tolerance |

**Table S5** List of DEGs related to photoperiodism, flowering (GO:0048573)

| Gene ID | Comparation | Log2 Foldchange | padj | Annotation |
| --- | --- | --- | --- | --- |
| Os01g0218500 | Heij-T *vs* Nipp-T | 5,281959 | 1,29E-27 | Phosphatidyl ethanolamine-binding protein (PEBP) family, flowering promotion (FTL) |
|  | Heij-O *vs* Nipp-O | 6,275449 | 3,28E-23 |  |
| Os02g0831800 | Heij-T *vs* Nipp-T | 1,126869 | 1,07E-14 | Homoserine kinase, homoserine/4-diphosphocytidyl-2-c-methyl-d-erythritol (CDP-ME) kinase family |
|  | Heij-O *vs* Nipp-O | 2,635081 | 2,37E-70 |  |
| Os06g0157500 | Heij-T *vs* Nipp-T | 3,557175 | 5,85E-14 | PEBP family, florigen, flowering time, Long-day promotion (RFT1) |
|  | Heij-O *vs* Nipp-O | 8,752413 | 3,44E-14 |  |
| Os06g0157700 | Heij-T *vs* Nipp-T | 8,563035 | 4,71E-86 | PEBP family, florigen, flowering promotion (HD3A) |
|  | Heij-O *vs* Nipp-O | 11,92518 | 4,44E-29 |  |
